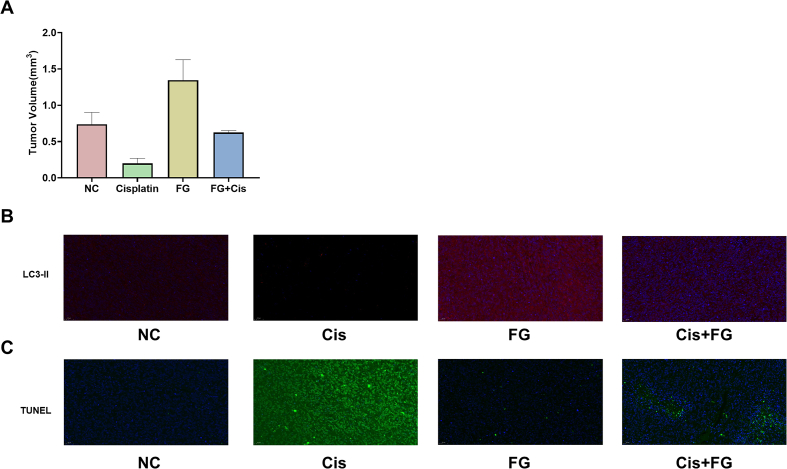# *EWSR1-PSMC5* fusion gene variously activating autophagy in drug resistance of osteosarcoma: A novel gene fusion model report and mechanism research

**DOI:** 10.1016/j.gendis.2024.101358

**Published:** 2024-06-21

**Authors:** Qing Pan, Wenbo Yang, Fuhua Huang, Wei Wu, Zengwu Shao, Zhicai Zhang

**Affiliations:** Department of Orthopaedics, Union Hospital, Tongji Medical College, Huazhong University of Science and Technology, Wuhan, Hubei 430022, China

Sarcoma is a kind of mesenchymal malignant tumor and often has poor chemotherapy response. There are many reasons for the insensitivity of sarcomas to chemotherapy, among which genetic changes are important. As a common type of gene alteration, gene fusion plays an important role in the pathogenesis and progression of tumors. In this study, we report a novel osteosarcoma-associated fusion gene, *EWSR1-PSMC5*, found in patients insensitive to chemotherapy. This gene is a novel fusion mode and has been found to play an important role in autophagy activation. The fusion gene may lead to the activation of autophagy through various signaling pathways, thus leading to the development of osteosarcoma resistance. We report this new fusion mode for the first time, and it should be noted that there is a less common report of *EWSR1*-related fusion gene in osteosarcoma.

Osteosarcoma is the most common primary malignant bone tumor. Currently, most patients with osteosarcoma undergo treatment involving a combination of neoadjuvant chemotherapy before surgery, followed by radical surgery, and then postoperative adjuvant chemotherapy. Clinical practice has demonstrated that the therapeutic effect of chemotherapy on osteosarcoma is limited, and many patients do not exhibit significant chemotherapy-induced necrosis, resulting in a poor prognosis for many. The reasons for osteosarcoma's insensitivity to chemotherapy are complex and may include genetic changes, immunosuppression, and alterations in the metabolic microenvironment. Changes in tumor genes often lead to the activation of oncogenes and the inactivation of tumor suppressor genes. These genetic alterations have been associated with resistance to chemotherapy and even targeted therapies. Therefore, the complexity of genetic variations in osteosarcoma complicates its diagnosis and treatment.

In some soft tissue malignancies, specific fusion genes frequently appear. These fusion genes often have functional roles and serve as targets for the development of new therapies. The *EWSR1-*related fusion genes, first identified in Ewing's sarcoma, include *EWSR1-FLI1* and *EWSR1-WT1*.^1^^,^^2^ These genes commonly promote tumor cell proliferation or contribute to chemotherapy resistance. In the case of chemotherapy-resistant osteosarcoma, we identified the expression of the *EWSR1-PSMC5* fusion gene. To date, this specific fusion gene has not been reported, and its function remains unknown. In this study, we demonstrated, both *in vitro* and *in vivo*, that the *EWSR1-PSMC5* fusion gene can contribute to osteosarcoma's resistance to chemotherapy by influencing autophagy and other related signaling pathways.

The patient, a 13-year-old female, was diagnosed with common osteosarcoma of the upper left humerus in 2018 and underwent neoadjuvant chemotherapy with cisplatin and other standard chemotherapy agents. The patient's surgery took place in January 2019, followed by continued postoperative adjuvant chemotherapy. The details of the specific treatment plan are provided in Table S1. Postoperative pathology reports indicated that the tumor necrosis rate reached 80% following neoadjuvant chemotherapy. DNA sequencing of the tumor tissue identified a fusion involving exons 1–16 of EWSR1 and exon 12 of PSMC5. The schematic representation of the fusion gene is depicted in Figure 1A. We demonstrated an increase in the transcription levels of the EWSR1-PSMC5 fusion gene in tumor tissue compared with peritumoral tissue using PCR (Fig. 1B).

**Figure 1:**
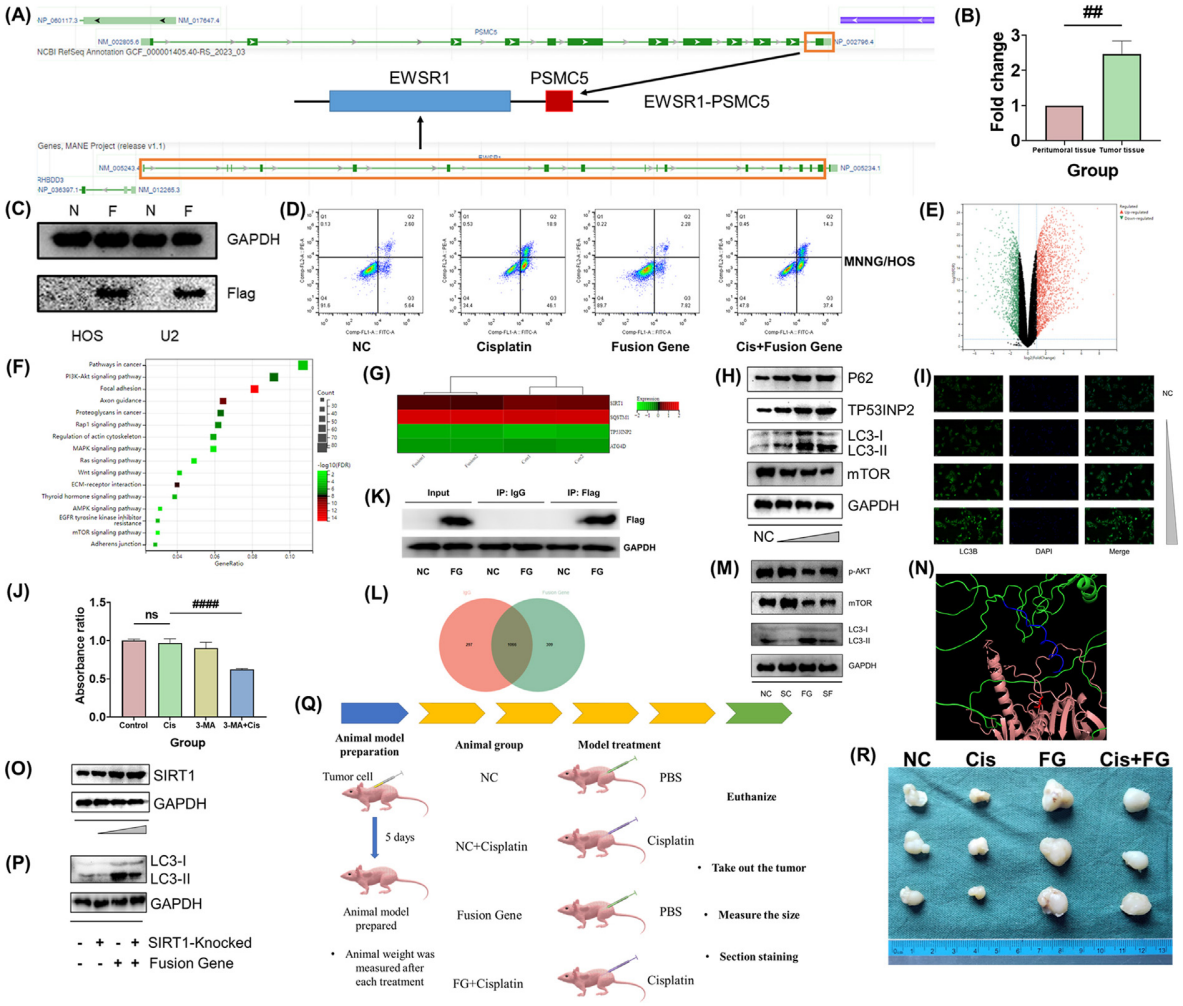
Fusion mode, expression verification, and functional exploration of *EWSR1-PSMC5* fusion gene. **(A)** Schematic chart of gene fusion patterns. **(B)** PCR was used to compare the expression levels of fusion genes in tumor and peritumoral tissue. **(C)** The expression levels of target genes in the fusion overexpression group and the NC group were compared by PCR and western blot. Figure C shows the expression of fusion genes in two osteosarcoma cell lines, with Flag as the label. **(D)** Resistance to chemotherapeutic drugs by fusion gene in the MNNG/HOS osteosarcoma cell line was confirmed by apoptotic flow cytometry. **(E)** Volcano plot of the distribution of different expression genes. **(F)** KEGG enrichment analysis of down-expression genes. **(G)** The expression levels of some autophagy-related genes, such as SIRT1, were shown by heat map. **(H)** Western blot analysis of autophagy-related markers. **(I)** Autophagy levels in osteosarcoma cells, demonstrated by immunofluorescence assay. LC3-II was used as a marker. **(J)** CCK-8 assay confirmed that 3-MA could block the protection of the fusion gene in two osteosarcoma cell lines. **(K)** The effect of fusion gene (FG) expression products captured by magnetic beads loaded with Flag protein antibodies. The Input group confirmed that the Flag was shown only in osteosarcoma cells with overexpression of the fusion gene. Magnetic beads loaded with IgG cannot capture the target protein. When the magnetic beads loaded with Flag anti-body interact with total proteins derived from osteosarcoma cells overexpressing fusion genes, the overexpressed products can be pulled down. **(L)** Venn plot represents the proteins bound to magnetic beads containing IgG and Flag antibody, respectively. **(M)** Western blot results showed that AKT inhibitor SC79 reduced the autophagy activation effect of the fusion gene. **(N)** LightDock software was used to analyze the interaction between fusion gene products and AKT protein. Analysis results indicated a possible effect of the *PSMC5* 12 exon in fusion gene construction on the phosphorylation of S473 in AKT. **(O)** Western blot assay confirmed the changes in SIRT1 expression level. **(P)** Autophagy level of osteosarcoma cells overexpressing fusion gene or NC was observed by inhibiting SIRT1 expression. **(Q)** Model scheme of *in vivo* experiments. **(R)** Tumor size display.

We confirmed the expression of the *EWSR1-PSMC5* fusion gene in two osteosarcoma cell lines using PCR and western blot assays (Fig. 1C; Fig. S1C–E). With CCK-8 assay and apoptotic flow cytometry, we observed that cell lines overexpressing the *EWSR1-PSMC5* fusion gene exhibited resistance to cisplatin and adriamycin (Fig. 1D; Fig. S1F–I). Further investigation through mRNA sequencing revealed 3344 differentially expressed mRNAs, illustrated in a volcano plot (Fig. 1E). These findings indicate a significant impact of the fusion gene on osteosarcoma cell behavior. Subsequently, GO cellular component, GO biological process, WikiPathways enrichment, and KEGG enrichment analysis of down-regulated expression genes were performed on differentially expressed mRNAs (Fig. 1F; Fig. S2A–C). We utilized PCR to validate the expression of SIRT1 and SQSTM1, confirming the accuracy of our mRNA sequencing results (Fig. S2D, E). The results of GO enrichment analysis showed the action location of the fusion gene expression product and the correlation with some biological processes that may be related to tumor growth, such as cell migration. KEGG and WikiPathways enrichment analysis showed that the overexpression of fusion genes was associated with the inhibition of the PI3K-AKT pathway and the inhibition of the mTOR pathway. We also found the high expression of some autophagy-promoting genes, such as SIRT1, indicating the activation of autophagy in osteosarcoma cell lines overexpressing *EWSR1-PSMC5* fusion genes (Fig. 1G). RNA sequencing results demonstrated increased expression of autophagy markers P62/SQSTM1, LC3, and TP53INP2, and decreased mTOR expression. These changes were further validated by western blot assays, showing a correlation between the fusion gene overexpression and the protein levels of P62, LC3-II, TP53INP2 (increasing trend), and mTOR (decreasing trend) (Fig. 1H; Fig. S3A–D). Immunofluorescence experiments confirmed that autophagy levels in osteosarcoma cells significantly increased with higher levels of fusion gene overexpression (Fig. 1I).

Using CCK-8 assay and flow cytometry, we found that osteosarcoma cell lines overexpressing the fusion gene exhibited increased resistance to cisplatin. However, after the administration of the autophagy inhibitor 3-MA, the sensitivity of the tumor cells to chemotherapy drugs was significantly enhanced, and the effect of the fusion gene on drug resistance was weakened (Fig. 1J; Fig. S4). Therefore, autophagy may play a key role in promoting drug resistance in osteosarcoma through the overexpression of the *EWSR1-PSMC5* fusion gene.

We further explored the relationship between the *EWSR1-PSMC5* fusion gene and enhanced autophagy. We detected the intracellular presence of the fusion gene via Flag protein expression, demonstrating this through the binding of magnetic beads coated with Flag antibodies to the fusion gene's expression products (Fig. 1K). Subsequently, we performed shotgun protein mass spectrometry and discovered that the expression product of the fusion gene could bind to 309 proteins, in contrast to IgG (control) (Fig. 1L). The increase in p-AKT content and the autophagy-promoting effect of the overexpressed fusion genes were mitigated using the AKT activator SC79 (Fig. 1M; Fig. S5F–H). Through protein interaction simulation analysis, we identified that the exon 12 portion of the *PSMC5* in the fusion gene may interact with the domain near the common AKT1 phosphorylation sites T308 and S473 (Fig. 1N; Fig. S5I).

In addition to affecting the classical AKT-mTOR signaling pathway, we observed a significant increase in SIRT1 levels in osteosarcoma cells overexpressing the *EWSR1-PSMC5* fusion gene, as evidenced by mRNA sequencing. This increase was further confirmed by western blot assays, which showed a correlation between the degree of SIRT1 expression and the extent of fusion gene overexpression (Fig. 1O; Fig. S6D). SIRT1 is known to mediate the deacetylation of certain proteins, such as those in the FOXO family, leading to metabolic changes. Importantly, we found that increased SIRT1 expression significantly correlated with the activation of autophagy. We compared the autophagy levels in osteosarcoma cells with SIRT1 overexpression to those with SIRT1 knockdown, revealing decreased autophagy levels in cells where SIRT1 was knocked down (Fig. 1P; Fig. S6E).

The flow chart of the animal experimentation process is depicted in Fig. 1Q. We observed an increase in tumor volume in the group with overexpressed fusion genes compared with the control group; however, the difference was not statistically significant. Nevertheless, resistance to cisplatin in the xenografts overexpressing the EWSR1-PSMC5 fusion gene was enhanced, as shown in Figure 1R and S7A. Data from these *in vivo* experiments further corroborated the role of the fusion gene in promoting drug resistance by elevating autophagy levels.

In this study, we identified the expression of the *EWSR1-PSMC5* fusion gene in osteosarcoma for the first time, thereby enhancing our understanding of *EWSR1*-related genes. We discovered that the product of the *EWSR1-PSMC5* fusion gene can reduce the expression level of AKT3 and can specifically bind to AKT, thereby inhibiting AKT's function. However, the decisive nature of this protein interaction remains a subject for further discussion. As AKT becomes non-functional, so does mTOR, leading to the activation of autophagy. This represents one of the most significant mechanisms of action of the *EWSR1-PSMC5* fusion gene. Moreover, the diversity of methods through which fusion genes activate autophagy is also evident in the promotion of autophagy through the activation of the non-classical, autophagy-related SIRT1-mTOR signaling pathway. In conclusion, the effect of the *EWSR1-PSMC5* fusion gene on autophagy activation is relatively well-established. Autophagy can protect tumor cells from the damaging effects of chemotherapy drugs.^3^ Nevertheless, this study has confirmed that even a moderate increase in autophagy levels can protect osteosarcoma cells from chemotherapy-induced damage.

The discovery of the *EWSR1-PSMC5* fusion gene in chemotherapy-resistant osteosarcoma presents a potential biomarker for predicting treatment responses, thereby facilitating personalized medicine approaches. This breakthrough lays the groundwork for the development of targeted therapies and combination treatments, focusing on the inhibition of the gene's function and associated pathways, including the modulation of autophagy. Further research into the mechanisms of this gene could pave the way for the development of new drugs and the repurposing of existing treatments.

It should be emphasized that the patient remains alive due to timely surgical intervention. While the patient underwent adequate adjuvant chemotherapy following surgery, it is the timely nature of the surgery, informed by pathological findings, that remains a critical factor in treatment efficacy. Our study suggests a strong correlation between chemotherapy resistance and the presence of the *EWSR1-PSMC5* fusion gene, shedding further light on the role of *EWSR1*-related fusion genes. In the future, this fusion mode should be noted across various tumors and studied in more detail.

## Ethics declaration

The Ethics Committee of Union Hospital, Tongji Medical College, Huazhong University of Science and Technology, approved this study, and informed consent was obtained from the patient. All animal procedures were reviewed and approved by the Institutional Animal Care and Use Committee of Huazhong University of Science and Technology (animal ethical number: 3799).

## Funding

This work was supported by the 10.13039/501100001809National Natural Science Foundation of China (No. 82072979).

## Conflict of interests

The authors declared no competing interests.

## Figures and Tables

**figs1:**
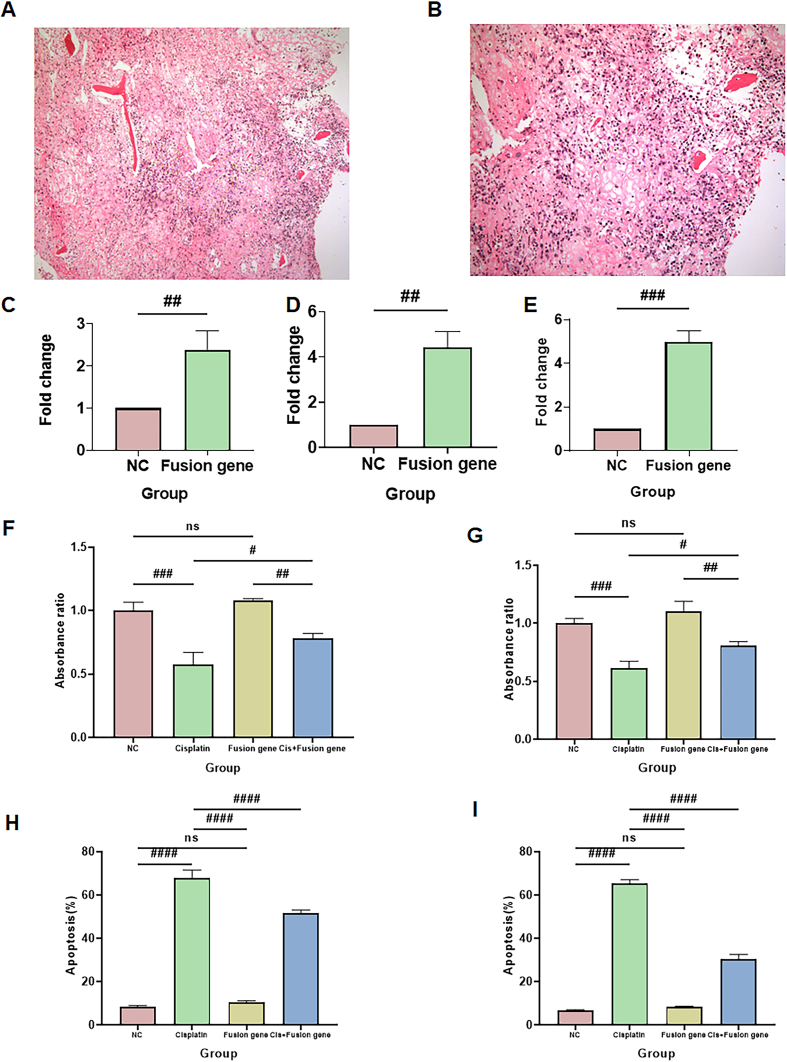


**figs2:**
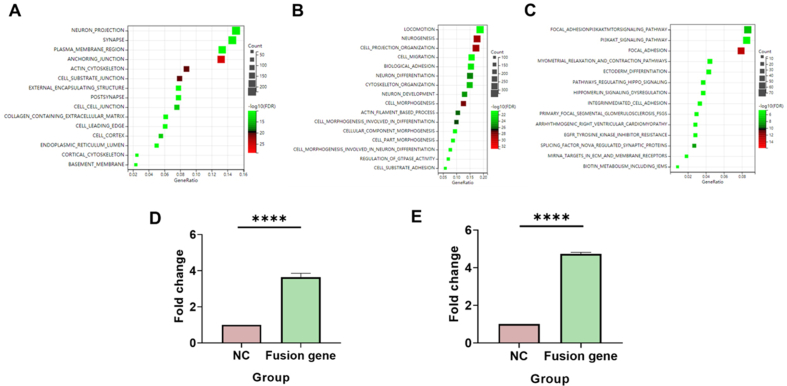


**figs3:**
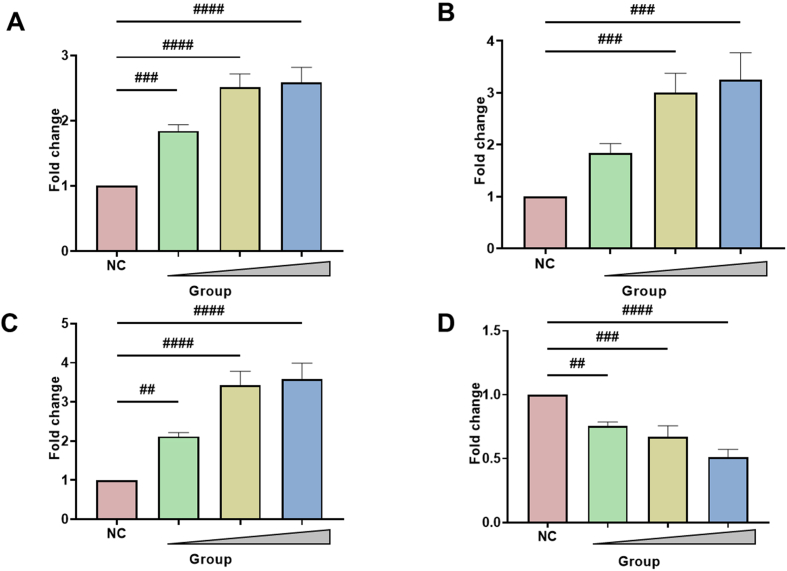


**figs4:**
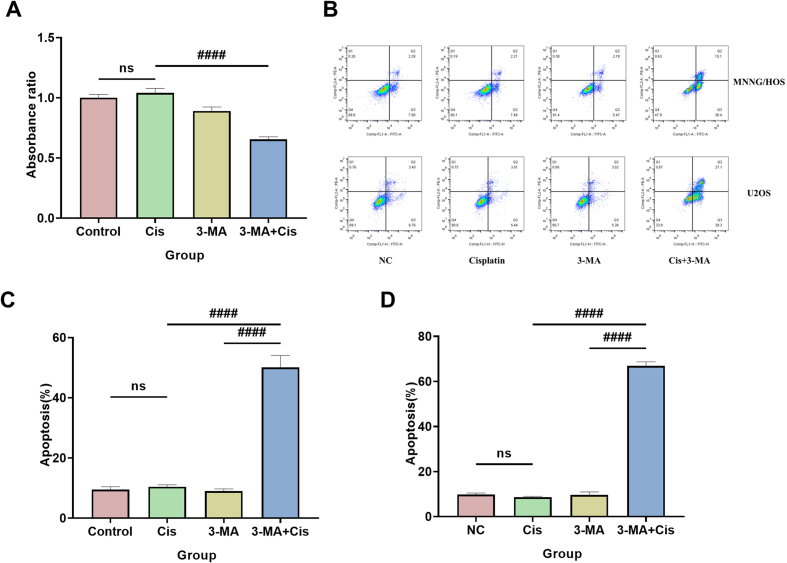


**figs5:**
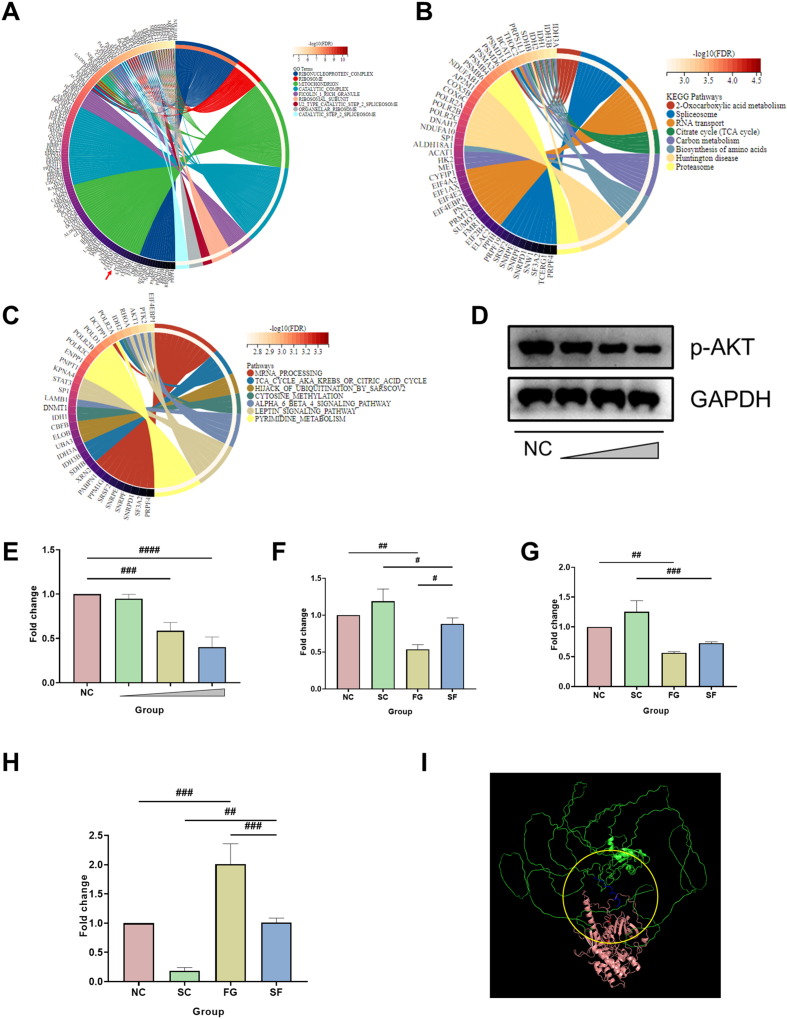


**figs6:**
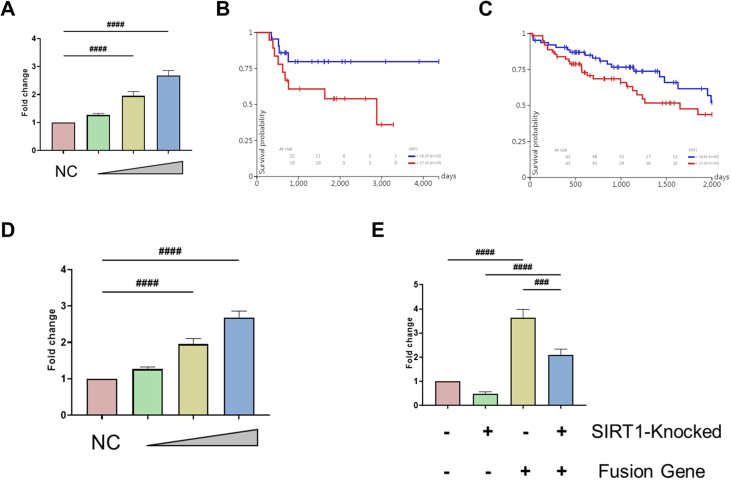


**figs7:**