# Supplementary material for: EWSR1-PSMC5 fusion gene variously activating autophagy in drug resistance of osteosarcoma: A novel gene fusion model report and mechanism research
Source: Genes Dis. 2024 Jun 21;12(3):101358. doi: 10.1016/j.gendis.2024.101358 (PMC11773463; doi:10.1016/j.gendis.2024.101358)
Supplement: Multimedia component 1 [file mmc1.docx]

**Methods**

**Cell lines culture and treatment**

Human osteosarcoma MNNG/HOS and U-2OS cell lines were purchased from Procell Life Science and Technology Co, Ltd. The cell culture medium was α-MEM/DMEM (High Glucose) + 10% fetal bovine serum + 100 U/ mL penicillin/streptomycin (finished medium, purchased from Procell). The cell culture environment was 37℃ with 5%CO_2_. In this study, the concentrations of cisplatin used to treat osteosarcoma cell lines are 3μM according to IC50.

**Colony-forming unit**

The cells were inoculated into 6-well plates with 750 cells per well. The inoculated cells were overexpressed with target genes or NC, according to experimental requirements. After blowing and beating evenly, the plate was placed in an incubator at 37℃ with 5% CO_2_. 3 days later, the medium containing the appropriate concentration of the drug (such as cisplatin) was replaced and the culture was continued. After 10 days, the medium was discarded, stained with 0.1% crystal violet and observed.

**Flow cytometry of apoptosis and ROS**

Annexin-V-FITC/PI apoptosis kit was selected to detect apoptosis. ROS detect kit was selected to detect apoptosis. We followed the instructions for the specific steps. The flow cytometry machine we used was the flow cytometer (BD Accuri C6 Plus). FLOWJO VX software was used to analyze flow cytometry results of apoptosis.

**Cell Counting Kit-8 (CCK-8) assay**

The cells in the culture flask were digested, suspended, and evenly planted in the 96-well plate. Each well contained 5000 cells. After cell adherence, the complete culture medium containing different concentrations of drugs was replaced. After co-culture, the cell viability was detected using the CCK-8 kit (Dojindo Laboratories, Japan). Each group was set with six replicates.

**Western blot analysis**

Sodium Dodecyl Sulfate-PolyAcrylamide Gel Electrophoresis (SDS-PAGE) was used to detect the expression of corresponding proteins in cells. The appropriate gel concentration was selected according to the molecular weight of the protein. First, Radio Immunoprecipitation Assay (RIPA) Lysis Buffer was used to lyse cells and extract proteins. The protein concentration was determined using a Bicinchoninic Acid Assay (BCA) protein concentration determination kit (Vazyme Biotech Co.,Ltd). Then, protease inhibitors and loading buffer with an appropriate volume were added, and the mixture was evenly mixed and heated at 95°C for 20 min to denature the protein. Then, sample loading and electrophoresis were performed. After electrophoresis, the Polyvinylidene Fluoride (PVDF) membrane was used for membrane transfer. We set the membrane transfer current as 200 mA and the membrane transfer time as protein molecular mass plus 10. After the membrane transfer, the 5% skimmed milk solution was used for blocking. The blocking condition was 1 h at room temperature. The PVDF membrane was then co-incubated with primary antibody at 4°C overnight. After incubation, the bands were washed with triethanolamine buffered saline solution with Tween 20 (TBST) solution and then co-incubated with secondary antibodies at room temperature for 1 h. After washing with TBST, the membrane was developed using the enhanced chemiluminescence (ECL) solution (Vazyme Biotech Co.,Ltd).

**RT-PCR**

The reagents used in the reverse transcription and amplification steps of RT-PCR are purchased from Takara Bio. RNA was extracted using TRIzol. According to the reagent instructions, add DEPC water, primer, template RNA, dNTP, Buffer, reverse transcriptase, etc. At the end of reverse transcription, amplified DNA was obtained and reverse transcriptase was inactivated. The next step is PCR. Template DNA, primer, dNTP, Mg^2+^, buffer, DEPC water and Taq enzyme were added into the system respectively. Set the temperature and time according to the instruction manual and complete denaturation, annealing and extension.

**mRNA sequencing methods and acquisition of genes with differential expression**

After the treatment, MNNG/HOS cells were collected and total RNA was extracted using TRIzol reagent (Invitrogen) for transcriptomic sequencing following the manufacturer's protocol. The groups of mRNA sequencing were set as fusion gene overexpression group and NC overexpression group. Illumina NovaSeq 6000 platform was used for sequencing. CASAVA base recognition was used to convert image data into reads, resulting in FASTQ files. The matrix of differentially expression genes was obtained through the processes of quality control, filtration, blast, calculation and differential expression analysis.

**Gene ontology (GO) enrichment analysis, Kyoto Encyclopedia of Genes and Genomes (KEGG) enrichment analysis**

After obtaining the gene sets with differential expression, we used the GO annotations of genes in the R software package org.Hs.eg.db (Version 3.1.0) and clusterProfiler (Version 3.14.3) for the GO enrichment analysis (21, 22). We used the KEGG REST API (https://www.kegg.jp/kegg/rest/keggapi.html) to obtain the latest KEGG pathway gene annotation. R software package clusterProfiler (Version 3.14.3) was used for enrichment analysis (21).

**Protein conformation and interaction analysis**

In this study, AlphaFold database was used to predict the structure of fusion gene fragments and target proteins(23, 24). The functional domains of AKT were identified through published studies. In this study, we focused on the effect of *PSMC5* exon 12 expression products on AKT. We focused on phosphorylation sites of AKT1 as a receptor. We used the LightDock tool for specific analysis(25). And Pymol 2.3.0 was used to plot the results(26).

**Protein qualitative assay (Shotgun)**

Protein qualitative assay was performed to verify which proteins the expression product of the fusion gene acted with. This experiment was divided into overexpressed NC and overexpressed fusion gene. The antibody of flag was used as a tool to capture fusion gene expression products. The interacting protein list was obtained by protein extraction, enzymatic hydrolysis, peptide desalination, mass spectrometry, blast and analysis processes.

**Animal experiment**

The subcutaneous tumor-bearing model of BALB/c-nude mice was used for the experiment. Each mouse was injected with 1.5 × 107/150 µL osteosarcoma cells near the groin of the right lower limb. The cells used included MNNG/HOS osteosarcoma cells that overexpressed NC or MNNG/HOS osteosarcoma cells that overexpressed fusion genes. The in vivo dose of cisplatin was 6mg/kg by intraperitoneal injection for a total of 3 times, with each interval of 2 days. Treatment was initiated when the tumor volume was about 50mm^3^. Two days after the final injection, the tumor was removed and measured volume and weight. TUNEL assay, Ki-67 assay and LC3-II immunofluorescence staining were performed. Relevant animal ethics documents were submitted to the school's animal ethics committee and verified.

[**Statistical**](javascript:;) [**analysis**](javascript:;)

The number of duplicates without special instructions is 3. We used analysis of variance to analyze the statistical difference between groups. p<0.05 indicated a statistically significant difference. We used GraphPad Prism 8 to conduct statistical tests and plot statistical figures. ANOVA analysis Was used as a statistical analysis method unless otherwise specified. In Figure, #: p<0.05; ##: p<0.01; ###: p<0.001; ####: p<0.0001.

**Results**

*EWSR1* has been reported to fuse with exons of many genes. We summarized the common phenomena of *EWSR1* fusion with other genes in soft tissue tumors as shown in Table S2.

**Figure legend of appendix**

**Figure S1**. **A, B.** Histologic sections of tumors in osteosarcoma patients with *EWSR1-PSMC5* high expression. The results showed that after chemotherapy, the tumor tissue presented necrosis, but there was still tumor growth; **C-E.** The expression levels of target genes in the fusion overexpression group and the NC group were compared by PCR and Western Blot. Figure C shows the PCR results, and Figure D, E shows the expression of fusion genes in two osteosarcoma cell lines, with Flag as the label. **F, G.** Resistance to chemotherapeutic drugs by fusion gene in two osteosarcoma cell lines, confirmed by CCK-8 assay; **H, I.** Resistance to chemotherapeutic drugs by fusion gene in two osteosarcoma cell lines was confirmed by apoptotic flow cytometry.

**Figure S2**. Gene expression profile enrichment analysis of osteosarcoma cells after fusion gene overexpression. **A.** GO enrichment analysis (Cellular Component) of down-expression genes; **B.** GO enrichment analysis (Biological process) of down-expression genes; **C.** WikiPathways enrichment analysis of down-expression genes. D, E. The PCR results for SIRT1 and SQSTM1 confirmed the fusion gene's resistance to chemotherapy drugs.

**Figure S3**. Expression levels of autophagy associated proteins in osteosarcoma cells overexpressing *EWSR1-PSMC5* and autophagy fluorescence levels in osteosarcoma cells. **A-D.** The statistical analysis of protein expression level of P62, TP53INP2, LC3-II, mTOR.

**Figure S4**. To verify the role of autophagy in *EWSR1-PSMC5* fusion gene. **A.** CCK-8 assay confirmed that 3-MA could block the protection of fusion gene in two osteosarcoma cell lines; **B.** Flow cytometry showed that 3-MA could block the protection of the fusion gene in two osteosarcoma cell lines; **C, D.** Statistical analysis of apoptotic flow cytometry.

**Figure S5**. Effect of *EWSR1-PSMC5* fusion gene expression products. **A.** GO enrichment analysis (Cellular Component) of proteins specifically bound to fusion gene expression products. Where AKT1 is marked with a red arrow; **B.** KEGG enrichment analysis of specific binding proteins of fusion gene expression products; **C.** WikiPathways enrichment analysis of specific binding proteins of fusion gene expression products; **D, E.** Western Blot results showed that with the increase of fusion gene expression level, the content of p-AKT decreased ;**F-H.** Western Blot results showed that AKT inhibitor SC79 reduced the autophagy activation effect of fusion gene; **I.** LightDock software was used to analyze the interaction between fusion gene products and AKT protein. Analysis results indicated a possible effect of the *PSMC5* 12 exon in fusion gene construction on the phosphorylation of S473 in AKT.

**Figure S6**. The effect of fusion gene expression products on autophagy through SIRT1-related pathways. **A.** PCR confirmed the increased expression level of SIRT1; **B, C.** The association between SIRT1 and poor prognosis of sarcoma (osteosarcoma) was confirmed by analysis of TARGET-OS and TCGA-sarcoma data sets; **D.** Western Blot assay confirmed the changes of SIRT1 expression levels; **E.** Autophagy level of osteosarcoma cells overexpressing fusion gene or NC was observed by inhibiting SIRT1 expression.

**Figure S7**. The effect of fusion gene *EWSR1-PSMC5* confirmed in vivo. **A.** Tumor size statistical chart; **B.** Immunofluorescence staining results of autophagy levels in different treatment groups. LC3-II as marker; **C.** TUNEL staining of tumor tissue.

**Table S1** Chemotherapy regimens used by patients and drugs used.

| Time | Chemotherapy regimens | Dosage^*^ and duration | Side effects |
| --- | --- | --- | --- |
| 2018/9/18 | MTX + DDP | MTX: 15.0 g, 1 day; DDP: 130 mg, 1 day. | None |
| 2018/10/10 | ADM + IFO + MESNA | IFO: 4.0 g, 2 days; ADM: 35 mg, 2 days; IFO: 3.0 g, 1 day. | None |
| 2018/10/30 | ADM + IFO + MESNA | IFO: 4.0 g, 2 days; ADM: 35 mg, 2 days; IFO: 3.0 g, 1 day. | None |
| 2018/11/20 | ADM + IFO + MESNA | ADM: 35 mg, 2 days; IFO: 4.0 g, 3 days. | None |
| 2018/12/19 | ADM+IFO+MESNA | ADM: 50 mg, 1 day; IFO: 3.5 g, 1 day; IFO: 4.0 g, 2 days. | Nausea and vomit |
| 2019/1/28 | HD MTX + DDP | MTX: 15.0 g, 1 day; DDP: 130 mg, 1 day. | None |
| 2019/2/19 | ADM + IFO + MESNA | ADM: 50 mg, 1 day; IFO: 3.5 g, 1 day; IFO: 4.0 g, 2 days. | None |
| 2019/3/15 | ADM + IFO + MESNA | ADM: 50 mg, 1 day; IFO: 3.5 g, 1 day; IFO: 4.0 g, 2 days. | None |
| 2019/4/8 | MTX + DDP | MTX: 15.0 g, 1 day; DDP: 130 mg, 1 day. | None |
| 2019/5/4 | ADM + IFO + MESNA | ADM: 50 mg, 1 day; IFO: 3.5 g, 1 day; IFO: 4.0 g, 2 days. | None |
| 2019/5/30 | ADM + IFO + MESNA | ADM: 50 mg, 1 day; IFO: 4.0 g，2 days; IFO: 3.0 g, 1 day. | None |
| 2019/6/25 | MTX + DDP | MTX: 15.0 g, 1 day; DDP: 130 mg, 1 day. | None |
| 2019/7/26 | ADM+IFO+MESNA | ADM: 50 mg, 1 day; IFO: 3.0 g, 1 day; IFO: 4.0 g, 2 days. | None |

Note: ^*^Medication frequency: once daily. MTX, methotrexate; DDP, cisplatin; ADM, adriamycin; IFO, isocyclophosphamide; HD, high dose.

**Table S2** Report of fusion genes associated with *EWSR1* in bone and soft tissue tumors.

| Tumor type | Chromosomal change | Gene fusion mode | Targeted pathway/gene | Clinical significance: Diagnosis, promotion, or inhibition of tumor proliferation | Reference |
| --- | --- | --- | --- | --- | --- |
| Small round cell tumors of connective tissue | t(11;22)(p13;q12) | *EWSR1-WT1* | SIK1 | Promotion | 31 |
|  |  |  | NTRK3 | Promotion | 32 |
| Peripheral neuroectodermal tumor | t(11;22)(q24;q12) | *EWSR1-FLI1* | TGF-β type II | Promotion | 33 |
|  |  |  | DDK-1 | Promotion | 34 |
|  | t(21;22)(q22;q12) | *EWSR1-ERG* | TGF-beta RII | Promotion | 35 |
|  | t(7;22)(p22;q12) | *EWSR1-ETV1* | TGF-beta RII | Promotion | 33 |
|  | inv(22)(q12;q12) | *EWSR1-ZSG* | UQCRH | Promotion | 36 |
| Mucinous/round cell liposarcoma | t(12;22)(q13;q12) | *EWSR1-DDIT3* | OPN, COL11a2 | Promotion | 37 |
| Angiomatoid fibrous histiocytoma | t(2;22)(q33;q12) | *EWSR1-CREB1* | IL-6 | Promotion | 38 |
| Clear cell sarcoma | t(12;22)(q13;q12) | *EWSR1-ATF1* | PRMT5 | Promotion | 24 |
| Extraosseous mucinous chondrosarcoma | t(9;22)(q22;q12) | *EWSR1-NR4A3* | PPARG | Inhibition | 39 |
| Ewing's sarcoma | t(11;22)(q24;q12) | *EWSR1-FLI1* | BRCA1 | Promotion | 40 |
|  |  |  | Core regulatory circuitry (CRC) | Promotion | 41 |
|  |  |  | DAX1(NR0B1) | Promotion | 42 |
|  |  |  | ATF4 | Promotion | 43 |
|  |  |  | GLI1 | Promotion | 44 |
|  |  |  | FOXO1 | Promotion | 45 |
|  |  |  | TNC | Promotion | 46 |
|  |  |  | SLFN11 | Promotion | 47 |
|  |  |  | ATG4B | Promotion | 48 |
|  |  |  | SPRY1 | Promotion | 49 |
|  |  |  | MRTFB/YAP-1/TEAD | Promotion | 50 |
|  |  |  | Casp3 | Promotion | 51 |
|  | t(21;22)(q22;q12) | *EWSR1*-ERG | Id2 | Promotion | 52 |
|  |  |  | TGF-beta RII | promotion | 35 |
|  |  |  | LAMB3 | Promotion | 53 |
|  | t(7;22)(p22;q12) | *EWSR1*-ETV1 | TGF-beta RII | Promotion | 35 |
